# The effectiveness of Multidimensional Treatment Foster Care for Preschoolers (MTFC-P) for young children with severe behavioral disturbances: study protocol for a randomized controlled trial

**DOI:** 10.1186/1745-6215-14-197

**Published:** 2013-07-05

**Authors:** Caroline S Jonkman, Carlo Schuengel, Robert Lindeboom, Mirjam Oosterman, Frits Boer, Ramon JL Lindauer

**Affiliations:** 1 Department of Child and Adolescents Psychiatry, Academic Medical Center, University of Amsterdam, Amsterdam, The Netherlands; 2 De Bascule, Academic Center for Child and Adolescent Psychiatry, Meibergdreef 5, Amsterdam 1105 AZ, The Netherlands; 3 Department of Clinical Child and Family Studies, EMGO Institute for Health and Care Research, VU University, Amsterdam, The Netherlands; 4 Division of Clinical Methods and Public Health, Academic Medical Center, University of Amsterdam, Amsterdam, The Netherlands

**Keywords:** Attachment, Autonomic reactivity, Behavior, Cortisol, Foster care, Preschool children

## Abstract

**Background:**

Among children placed out of home, behavioral and relationship functioning is often problematic. When placed in foster care, problems tend to persist or even worsen and increase the risk of placement breakdown. Multidimensional Treatment Foster Care for Preschoolers is an intensive behavior-focused program for young foster children (3 to 7 years) aiming to provide children with a positive and stimulating foster family setting and individually tailored behavioral interventions. This study will be the first to examine the effectiveness of Multidimensional Treatment Foster Care for Preschoolers outside the US and to examine the effectiveness across a broader range of problems related to foster care.

**Methods:**

This is a randomized controlled trial, wherein we expect to include 80 child-foster carer dyads. Forty dyads will be assigned to Multidimensional Treatment Foster Care for Preschoolers and 40 to treatment as usual, following pre-randomization. Data to be gathered concern problem behavior, symptoms of attachment disorder, post-traumatic stress symptoms, quality of life, hypothalamic-adrenal-pituitary axis functioning, parental stress and autonomic reactivity, to be collected via questionnaires, observations, interviews, saliva and recording at six time-points over 24 months. To compare treatment outcomes, Fisher’s exact tests and repeated measures (mixed models) and independent t-tests will be used. All analyses will be performed following the intention-to-treat principle.

**Discussion:**

Examining the generalizability of previous findings in the US and extending these previous findings is a step towards improving knowledge about treatment of young foster children with severe behavioral, emotional and attachment problems.

**Trial registration:**

NTR1747

## Background

The large proportion of young children in the foster care population is of concern, with children younger than five years old representing over a third of foster children in the US (33% of 400,540 [1]) and the Netherlands (36% of 8,944 [2]). Children often enter foster care after serious neglect and abuse. Among foster children, there is wide variation in behavioral and relationship functioning, which is on average more problematic compared with that of children living with biological parents [3-5]. Foster children often suffer from post-traumatic stress disorder [6] as well as more complex traumatic symptoms [7], more disorganized attachment [8], and high frequencies of clinical symptoms of disturbed attachment [9-11]. Additionally, adverse or inconsistent caregiving tends to cause atypical functioning of children’s biological stress systems, such as changes in the hypothalamic-adrenal-pituitary (HPA) axis, represented by abnormal cortisol segregation [12,13], and altered stress regulation activity of the autonomic nervous system (ANS) [14]. After placement in foster care, problems tend to persist or even worsen [15,16], and not only negatively affect the parenting behavior of foster carers [17] but also increase the risk of placement breakdown. Placement breakdown may start a vicious circle in which the chance of another failure increases with every breakdown [18]. The last alternative is often residential placement, which fails to provide opportunities for developing attachment [19], causes developmental delays [20], and places children at further risk. To prevent foster children from further problems, these children are in need of evidence-based programs that combine foster placement with effective treatment of emotional and behavioral disorders and parental stress. Multidimensional Treatment Foster Care for Preschoolers (MTFC-P) tends to address these needs, providing a positive and stimulating foster family setting for these children. Foster caregivers are taught effective behavioral management strategies and children receive individually tailored behavioral interventions. Dozier and Rutter [21] suggested that when foster carers and children gain control over behavioral problems, the potential for the development of a (secure) attachment relationship increase. The absence of behavioral problems results in less parental stress for the foster carers, allowing them to better respond to the child in a sensitive and nurturing way - supporting children’s belief in the caregiver’s availability. With the increase of (secure) attachment behaviors, the risk of disturbed attachment declines, because behaviors of either type are theoretically incompatible with one other [11,19].

Previous studies in the US have shown that, relative to children in regular foster care, children in MTFC had fewer placement failures [22], improved HPA axis functioning [23], increased secure attachment behavior and less resistant behavior [24]. Although MTFC-P is quite successful in the US and transportability of the MTFC model for older children has been shown in a Swedish context [25], the efficacy of the preschool version has not been replicated in other countries where implementation challenges and cultural differences may play a role. Outside the US, in the Netherlands, only a small pilot study was conducted, preliminary to this study. Early findings suggested less problem behavior in the MTFC-P intervention [26].

This protocol is for a study intending to examine whether US findings regarding MTFC-P also apply for children in foster care in the Netherlands. The study also aims to extend on previous studies by examining the effects of MTFC-P on post-traumatic stress symptomatology, symptoms of attachment disorder and quality of life. With the present study, we compare the effects of MTFC-P with a treatment as usual (TAU) for foster children at risk for placement breakdown. We hypothesize that MTFC-P is more effective than TAU for young foster children with emotional and behavioral disorders. Effectiveness is measured in terms of decreased problem behavior; fewer symptoms of disturbed attachment; fewer severe post-traumatic stress symptoms; improved quality of life; recovery of HPA-axis functioning, indicated by recovery of atypical cortisol activity; and less parental stress. We expect that, relative to control children, at the end of the treatment children in the MTFC-P group will show less maladaptive responses of the ANS, indicated by heart rate, respiratory sinus arrhythmia and pre-ejection period, to separation and reunion with caregivers and strangers.

## Methods

### Participants

Eligible participants are children between 3 and 7 years, referred to our department of Treatment Foster Care at De Bascule, Academic Center for Child and Adolescent Psychiatry (Amsterdam, Netherlands). Eighty child-foster carer dyads will be included in the study.

### Inclusion criteria

The inclusion criteria for children and foster carers are to meet the eligibility criteria of MTFC-P. Children are eligible if they are between 3 and 7 years of age and indicated for permanent foster care placement.

### Exclusion criteria

Children are excluded if they do not meet the inclusion criteria and when foster parents provide no informed consent. When children’s legal authorities provide no informed consent or when foster parents disagree with the treatment they are assigned to, the children are excluded from the randomized trial.

### Intervention

The TAU is an intensive treatment for foster children from 0 to 18 years, aiming to prevent placement breakdown. TAU comprises a 3-month diagnostic phase and 9-month treatment phase, delivered by a multidisciplinary team (system therapist, developmental psychologist, child psychiatrist and social worker). Outcomes of the diagnostic phase direct the treatment that will be provided, for example, trauma therapy, parent–child interaction therapy, individual therapy, medication or a combination of these. In addition, during the treatment phase, foster carers receive two-weekly coaching from a social worker, to enhance parental skills and to provide the foster carers with support. They also participate in systemic family therapy sessions, together with children and other important family members.

MTFC-P is an intensive behavior focused program for young foster children (3 to 7 years of age), aiming to decrease children’s problem behavior and increase social behaviors to promote further placement stability. For 9 months, children are placed with therapeutic foster carers. Therapeutic foster carers are highly trained foster carers who work as semi-professionals closely with MTFC-P staff. MTFC-P is delivered through a treatment team approach, by a specially trained MTFC-P team led by a program supervisor. Children receive individual training and weekly therapeutic playgroup from a skill trainer. Therapeutic foster carers participate in weekly group meetings and receive frequent home visits and ongoing support from a foster carer consultant. A family therapist supports important members of the biological family. Children receive behavioral interventions that are based upon Patterson’s theory of coercion with its principles of social learning [27], to stimulate pro-social behavior and diminish behavioral problems. A key notion is that behavioral problems result from reinforcing negative behavior and lack of modeling of positive behavior. To tackle this, MTFC-P makes use of two principal techniques. First, skills trainer and therapeutic foster carers reward positive behavior. Second, therapist and foster carers ignore negative behavior; instead, they offer an alternative or put the child on a short time-out from contact. Therapeutic foster carers are responsible for the continuity of children’s behavioral interventions. To maintain a beneficial treatment setting for children, therapeutic foster carers are therefore encouraged to stay consistent and responsive toward the child. In addition, they receive parental strategies to promote positive behavior and effective non-abusive limit setting for problem behavior [28]. After the initial 9 months, children are transferred to an aftercare setting. Here, the skills trainer continues children’s training and foster parents (or birth parents in case of reunification) receive parenting practices for approximately 3 months. Although the US aftercare setting is an adoptive family or the birth family, in the Netherlands children are then placed in a permanent foster family or the birth family. Adoption of foster children in the Netherlands is rare, as the primary aim of foster care services is reunification with the birth parent. Furthermore, because after MTFC-P children no longer need intensive treatment, it is not intended that children stay with the therapeutic foster carers. These trained foster carers will adopt a new child for 9 months.

### Measures

Measures are directed towards primary and secondary objectives. Data are gathered from primary foster carers, children and teachers.

### Primary objectives

#### Problem behavior

The Child Behavioral Checklist for ages 1.5 to 5 (CBCL 1.5-5 [29]) and 6 to 18 (CBCL 6–18 [30]) assesses the internalizing and externalizing behaviors of children. The CBCL is completed by foster parents and consists of hundred (ages 1.5-5), respectively 113 (ages 6–18) items. Items are rated on a 3-point scale (*0 = not at all true, 1 = somewhat true, 2 = very true*). A Dutch translation of the CBCL is used. Prior studies in Dutch populations revealed satisfactory reliability and validity of the CBCL 1.5-5 and 6–18 [31,32]. Preliminary analyses based on data from the present study revealed good internal consistency with alphas >0.80 for all scales except for internalizing problems (alpha = 0.60).

The Teacher Report Form for ages 1.5 to 5 (TRF 1.5-5 [29]) and 6 to 18 (TRF 6–18 [30]) assesses children’s school functioning and behavioral problems. The TRF is completed by foster parents and consists of hundred (ages 1.5-5), respectively 113 (ages 6–18) items. Items are rated on a 3-point scale (*0 = not at all true, 1 = somewhat true, 2 = very true*). Depending on the age of study children, daycare providers or teachers complete this questionnaire. Preliminary analyses based on data from the present study revealed good internal consistency with alphas >0.80 for all scales except for internalizing problems (alpha = 0.44) and externalizing problems (alpha = 0.58). A Dutch translation of the TRF is used. Psychometric properties have been found to be adequate in Dutch populations [33].

The Strengths and Difficulties Questionnaire (SDQ [34]) is a behavioral screening instrument, completed by (foster) parents. This questionnaire consists of 25 items, rated on a three point scale (0 = not true, 1 = somewhat true, 2 = very true) testing children’s psychological attribution to five subscales and one total scale (alphas given in parentheses): emotional problems (0.65), conduct problems (0.68), hyperactivity (0.77), peer relations (0.57), prosocial behavior (0.73) and total difficulties (0.67). Alphas were based on preliminary data from the present study. We use a Dutch translated version of the SDQ. Reliability and validity of the SDQ-parent report in a Dutch sample have been found to be satisfactory [35].

The Parent Daily Report (PDR [36]) is a telephone interview with foster carers and is conducted daily during weekdays. It assesses the occurrence of 38 problem behaviors (for example, cruelty to animals, arguing) within the past 24 hours, which are scored on a two-point scale (0 = not occurred, 1 = occurred at least once). The PDR has previously been used as a measure for treatment outcomes; psychometric properties have been found to be adequate [37]. The PDR is translated into Dutch.

#### Attachment

The Disturbance of Attachment Interview (DAI [38]) is used to assess symptoms of reactive attachment disorder [6]. Twelve items of the DAI indicate symptoms of inhibited attachment (five items) or disinhibited attachment (three items) and secure base distortions (four items). Items are coded 0 if the symptom is definitely not present, 1 if there is some evidence for the symptom and 2 if the symptom is definitely present. Criteria for a reactive attachment disorder classification is a score of 2 (symptom definitely present) on one of the items of the subscales. Based on preliminary data, the intraclass correlation (ICC) for single measure (two-way random effects) was estimated based on the degree of agreement between the two interviewers, for the subscale Inhibition (ICC (95%) = 0.83), Disinhibition (ICC(95%) = 0.86) and Secure Base Distortions (ICC(95%) = 0.79). Previous research has revealed acceptable validity, internal consistency and satisfactory inter-rater reliability [39,40].

The Strange Situation Procedure [41] is a laboratory observation. During this separation and reunion procedure, the quality of the attachment relationship between children and foster carers is assessed. We use a modified procedure, according to the guidelines of the Preschool Attachment System [42,43], adding episodes 7 and 8 (see Table 1). One of the developers (Dr Robert Marvin) trained the researchers in the coding system.

**Table 1:** Episodes in the strange situation procedure

**Episode**	**Duration (minutes)**	**Description of episode**
1	-	Parent and child are in the room
2	3	Stranger enters the room
3	3	Parent leaves the room (separation parent 1)
4	3	Parent returns (reunion parent 1)
5	3	Stranger leaves the room (separation stranger 1)
6	1½	Parent leaves the room (separation parent 2)
7	3	Stranger enters the room (reunion stranger 1)
8	1½	Stranger leaves the room (separation stranger 2)
9	3	Parent enters the room (reunion parent 2)
10	3	Stranger enters the room (reunion stranger 2)

### Secondary objectives

#### Post-traumatic stress symptoms

The Trauma Symptom Checklist for Young Children (TSCYC [44,45]) determines the extent and type of post-trauma symptoms based on the (foster) parent’s report. Foster carers report how often each experience (90 items) happens to their child on a four point scale (1 = not at all, 2 = sometimes, 3 = often, 4 = very often). The psychological impact of negative life events in children is categorized by the nine clinical scales of the TSCYC (alpha, based on preliminary data from the present study is given in parentheses); anxiety (0.71), depression (0.75), anger (0.89), post-traumatic stress intrusion (0.73), post-traumatic stress avoidance (0.78), post-traumatic stress hyperarousal (0.79), post-traumatic stress total (0.86), dissociation (0.88) and sexual concerns (0.56). A Dutch translation of the TSCYC is used. The TSCYC demonstrates good reliability [44], and moderate convergent and discriminant validity [46].

#### Autonomic nervous system responses

The VU University Ambulatory Monitoring System (VU-AMS [47]) is a lightweight ambulatory device measuring ANS responses that is used during the Strange Situation Procedure. To obtain continuous electrocardiogram and impedance cardiogram readings, representing ANS responses, a researcher places seven disposable electrodes (Conmed Huggables® Pediatric/Neonatal Electrodes (ConMed Corporation, Utica, N.Y., USA) on the child’s upper body. To minimize influences of physical activity, the child is placed on a chair behind a desk with toys. The heart rate and respiratory sinus arrhythmia, as indicators of parasympathetic influences, and the pre-ejection period, as an indicator of sympathetic influences, are extracted from the VU Data, Analysis & Management Software (VU-DAMS) program. Heart rate is automatically extracted from VU-AMS in beats per minute. Respiratory sinus arrhythmia, defined as the variability in heart rate based on differences between the largest and shortest inter-beat intervals in milliseconds during respiration, is automatically calculated by the VU-DAMS. Furthermore, VU-DAMS calculates the time interval between the electrocardiogram Q-wave (onset of ventricular depolarization) and the impedance cardiogram B-point (onset of left ventricular ejection of blood in the aorta), indicating the pre-ejection period.

#### Hypothalamic-adrenal-pituitary axis functioning

Salivary cortisol is collected to determine recovery of children’s HPA functioning and foster carers HPA axis functioning over the course of the intervention. To measure basal diurnal cortisol patterns, we collect salivary samples in the early morning (immediately after waking), morning (waking plus 30 minutes) and evening from children and foster carers on five consecutive weekdays. Salivary cortisol collection is scheduled 3, 6 and 9 months after the start of treatment. The samples are obtained with a cotton collection device (Salivette; Sarstedt, Rommelsdorf, Germany). Children and foster carers received verbal and written age-appropriate instructions for saliva collection. Foster carers are instructed to assist children in chewing the swab for one minute and not to touch the tube when placing it back into the tube. Tubes are stored for a maximum of 6 days in the respondent’s refrigerator and then stored in −18°C at the research institute. All of an individual’s samples are analyzed at once, to minimize within-subject variation. Duplicate analyses are performed by the Cortisollabor in Trier (University of Trier, Trier, Germany), using a competitive solid phase time-resolved fluorescence immunoassay with fluorometric endpoint detection. Duplicate samples with coefficients of variation lower than 10% (values over 5 nmol/L) or 15% (values between 2 and 5 nmol/L) are re-analyzed.

#### Parenting stress

The Nosi-K [48] is a Dutch translation and short version of the Parenting Stress Index [49]. This instrument screens for stress related to the caregiver-child relationship and consists of 25 items, rated on a six point scale (1 = totally disagree, 2 = disagree, 3 = somewhat disagree, 4 = somewhat agree, 5 = agree, 6 = totally agree). The complete Nosi questionnaire has Cronbach’s alpha between 0.63 and 0.97 and moderate concurrent, discriminant and criterion validity [48,50].

#### Quality of life

The kiddy-KINDL [51] is a questionnaire to measure health-related quality of life in young children (4 to 7 years). Kiddy-KINDL parent version consists of 24 items, over six dimensions: physical well-being, emotional well-being, self-esteem, family, friend and everyday functioning. Items are rated on a five point scale (1 = never, 2 = rarely, 3 = sometimes, 4 = often, 5 = all the time). The KINDL has good psychometric qualities [51]. We used a Dutch translation of the kiddy-KINDL.

### Sample size calculation

Sample size is calculated based on the DAI (see Measures). The DAI is a dichotomous measure reflecting symptoms of disturbed attachment (1 = not or somewhat present, 2 = definitely present). From cross-sectional data it is known that the prevalence of symptoms of disturbed attachment in comparable populations is approximately 40% [39]. Based on unpublished pilot data, which also suggested 40% of participants would be symptomatic at any time-point in the TAU intervention, we expect that in the present randomized controlled trial (RCT), a 40% level of symptomatic individuals will be maintained until the end of the TAU. Based on Fisher and Kim’s study [24], which reported increased secure attachment behavior (behavior that is incompatible with disturbed attachment) after MTFC-P, we expect a decline of disturbed attachment over the course of the MTFC-P. The aim is to reduce the proportion of children with symptoms of disturbed attachment to 5% at the end of MTFC-P. Including 34 children in both conditions can statistically reveal a difference between conditions with 90% power, using Fisher's exact test with a 0.05 two-sided significance level for analysis. The study was initially planned as a ‘normal’ pre-randomization trial. However, we observed low recruitment of participants because children arrive in a crisis situation and obtaining consent from biological parents was problematic (see below, Randomization). We therefore changed to a pre-randomization with single consent method to allow the birth parents enough time to decide on their participation. Parents of children randomized to MTFC-P who refused consent received TAU after randomization. We expected a 10% cross-over rate. This cross-over rate reduces the efficiency of the trial by 19% and requires a 20% addition to the sample size [52]. We therefore augmented the planned sample size from 34 to 40 per treatment arm (under 90% power) to counter the dilution effects by the cross-overs in the intention-to-treat analysis and to diminish the type II error probability.

### Procedure

#### Recruitment

Eligible children are recruited from the department of Treatment Foster Care at De Bascule, Academic Center for Child and Adolescent Psychiatry (Amsterdam, Netherlands). Children’s legal authorities and foster carers receive information about the study. After approximately one week, a researcher contacts them and asks if all the information is understood. When children’s legal authorities or foster carers are interested in participation, an appointment is made to sign explicit informed consent.

#### Randomization

Random allocation to either condition is needed to reduce selection bias and to be able to attribute effects to MTFC-P or TAU. However, referral to foster care is often made in crisis and immediate start of care is a prerequisite. The start of foster care is also usually accompanied by resistance or absence of the biological parent (the child’s legal authority) or a vacuum in authority. We expect this brief time interval between referral and start of the intervention, together with inaccessibility of a legal authority, to hamper random allocations. Following conventional randomization, researchers are unable to ensure that the child’s legal authority has retrieved, understood and signed informed consent before the child must be allocated to a treatment [53]. This results in a preliminary loss of participants. A pre-randomization trial, wherein randomization is determined prior to informed consent [54], is implemented as this promotes the inclusion of participants by extending the time period between referral and start of treatment. Researchers then have more time to obtain informed consent, avoiding preliminary dropout [55]. An independent researcher makes a random allocation list, varying per time period. Distribution of assignment to both conditions varies over time periods (approximately every six months), based on available treatment places. This is necessary to guarantee that children receive immediate treatment and are not placed on a wait list. Conditions are disclosed to the treatment team and participants immediately after referral. If legal authorities will not sign informed consent, children receive TAU. The condition is not disclosed to any members of the research team concerned with coding data.

#### Data collection

Data is collected at six time-points over 24 months: T(1) at start, T(2) 3 months after start, T(3) 6 months after start, T(4) 9 months after start (end of treatment), T(5) 12 months after start and T(6) 24 months after start. Parents and teachers are asked to fill out the questionnaires at all six measurements occasions. Questionnaires are sent to their homes, but when necessary researchers will help them filling out the questionnaires by telephone or face-to-face. The DAI interview is administered by telephone at T(2) and T(4) by a trained interviewer. Saliva is collected at T(2), T(3) and T(4) on five consecutive days, three times a day (see Measures). During the saliva collection, foster carers are called everyday to complete the PDR. The Strange Situation Procedure and accompanying VU-AMS measure is scheduled one week before the end of the treatment (see Table 2).

**Table 2:** Research procedure

**Study phase**	**Time-point**	**Time after start**	**Action**
**Recruitment**			Assess for eligibility
			Randomization
			Send information
			Complete informed consent^a^
**Treatment**	T(1)	6 weeks	Questionnaires
	T(2)	3 months	Questionnaires, interview, saliva collection
	T(3)	6 months	Questionnaires, saliva collection
	T(4)	9 months	Questionnaires, interview, saliva collection, observation, autonomic nervous system recording
**Follow-up**	T(5)	12 months	Questionnaires
	T(6)	24 months	Questionnaires

^a^ No consent – child randomized to treatment as usual.

#### Blinding

Researchers coding the interviews and observations are blind to group allocation. The condition is disclosed to participants immediately after referral.

#### Planned statistical analyses

Analyses are performed using the software package SPSS (Statistical Package for the Social Sciences), version 17.0. Fisher’s exact tests are used for the categorical data, effect sizes are estimated by the relative risks, absolute risks and relative risk reduction. Effect parameters are presented within a 95% confidence interval. The continuous measures are analyzed with repeated measures (mixed models) and independent t-tests. Effect sizes are indicated by partial eta squared and Cohen’s d. Primary analyses are performed following the intention-to-treat principle. However, we will also perform a ‘treatment received’ analysis and a separate comparison of children whose parents refused participation and who received TAU after randomization to MTFC-P. Depending on the amount and type of data missing we will explore possibilities to deal with missing data, but we plan to use mixed models.

### Medical ethical approval

The study (METC 09/046) is approved by the AMC - Medical Ethical Committee (Academic Medical Center Amsterdam, The Netherlands; April, 2009).

### Data privacy

Data gathered during the study are treated as confidential. Participants are given a number; a document with the combined names and numbers is only available for members of our research team. Data are processed using the participants’ numbers, names are omitted and data is stored in lockable cabinets. Research reports will not mention names or other information that can be linked by others to the individual participants. Researchers declare that they will not disclose any participant information to third parties without permission.

### Publicity policy

Study findings will be submitted to international peer-reviewed journals and presented at international scientifically orientated conferences. Policy makers and service providers in the Dutch mental health care are informed about the study findings through national conferences.

## Discussion

This article describes our study protocol for examining the effectiveness of MTFC-P for young foster children with behavioral, emotional and attachment disorders. The experimental condition, MTFC-P is compared with treatment as usual using randomized allocation. Relevance for this study derives from a shortage in evidence-based programs combining foster care placements with effective treatment of behavioral, emotional and attachment disorders, while the population of young foster children is still growing and problems become more taxing [56]. Various treatment foster care programs have been developed across the world, many lacking a clear treatment protocol or theoretical framework. This partly explains why only a few, diverse and small-scale studies on treatment foster care programs have been published. Although these studies have documented positive outcomes, the generalizability of their outcomes remains uncertain [57]. Fisher’s study [13,22-24,57] is one of the first to examine the effectiveness of foster care placement with active treatment of emotional and behavioral disorders using random allocation. Although MTFC-P is proven successful in the US, its efficacy has only been tested by its developers and not been replicated by other researchers and in other countries where cultural differences may play a role and TAU is country and institution specific. This study will be the first to examine the effectiveness of MTFC-P outside the US and the first to examine the effects of MTFC-P on post-traumatic stress symptomatology, symptoms of attachment disorder and quality of life. A small pilot investigation [26] suggested promising outcomes, with fewer problem behaviors over the course of the intervention. Because this pilot was an uncontrolled and non-randomized trial, the present study was initiated as an addition to it.

Including sufficient participants using random allocation is a major challenge in this study. Following conventional randomization we do not expect researchers to be able to ensure that the child’s legal authority signs informed consent before the child must be allocated to a treatment [53]. Referral to foster care is often made in crisis and immediate start of care is a prerequisite. Concurrently, start of foster care is usually accompanied with resistance or absence of the biological parent (the child’s legal authority) or a vacuum in authority. We expect that this brief time interval between referral and start of the intervention together with inaccessibility of a legal authority will hamper random allocations and causes preliminary loss of participants. A pre-randomization trial, wherein randomization is determined prior to informed consent, is implemented as we expect this design to promote random allocation and improve inclusion of participants [55] as it extents the time period between referral and start of the treatment. Researchers therefore have more time to obtain informed consent, avoiding preliminary dropout. However, there is a serious statistical drawback to using pre-randomization according to Zelen, through participant crossover to TAU [52,54]. Crossover would also occur in a normal post-randomization RCT but is likely to be greater in the Zelen design because participants that refuse one of the treatments of interest would be excluded from participation in a normal RCT. These crossovers introduce dilution bias that will lead to an underestimate of the ‘true’ treatment effect in the intention-to-treat analysis. To cope with this dilution effect, we increased the sample size correspondingly to reduce the likelihood of a type II error. Advantages of the Zelen design are that it is closer to normal clinical practice because the allocated treatment is discussed with the parent who may or may not refuse the treatment on offer. This also results in a more representative sample of participants because, within a usual RCT, refusal of consent usually leads to a trial sample that is different from the general treated population. We therefore will also perform a ‘treatment received’ analysis in addition to the planned intention-to-treat analysis to represent the more natural state of affairs that results from pre-randomization.

A second limitation to our RCT is the lack of an adequate baseline measure. Because children are new in the foster family at the start of the intervention, we decided to postpone the first assessment. A new placement is often accompanied by a temporary increase or decrease in problems, and foster carers and children have just met. Adhering to a randomized trial will minimize baseline differences between children in the two conditions. A third limitation is that foster parent’s perception of children’s behavior is one of the primary focuses of the treatment, but also a primary objective of the present study. We attempt to limit the effect of reactive measures by the inclusion of teachers’ perceptions of children’s behavior. In addition, we inserted the Strange Situation Procedure. Although this observation of attachment is only post-treatment, we expect that the random allocation will diminish selection bias. Other alternatives have been considered, such as observational measures of children’s behavior, but their benefits are not expected to compensate for the extra burden they would place on participants. If we are able to meet the challenges and include sufficient participants, we will generate knowledge about the effectiveness of an intensive behavioral focused program, developed to gain stability of placement for young foster children.

## Status of the trial

The study started in January 2009. After approval by our Medical Ethical Committee, we started recruiting mid-2009 and inclusion is still in progress. We intend to publish study results at the end of 2013.

## Abbreviations

ANS: Autonomic nervous system; CBCL: Child Behavioral Checklist; DAI: Disturbance of Attachment Interview; HPA: Hypothalamic-adrenal-pituitary; ICC: Intraclass correlation; MTFC-P: Multidimensional Treatment Foster Care for Preschoolers; PDR: Parent Daily Report; RCT: Randomized controlled trial; SDQ: Strengths and Difficulties Questionnaire; TAU: Treatment as usual; TRF: Teacher Report Form; TSCYC: Trauma Checklist for Young Children; VU-AMS: VU University Ambulatory Monitoring System; VU-DAMS: VU Data, Analysis & Management Software.

## Competing interests

The authors declare that they have no competing interests.

## Authors’ contributions

RJLL obtained funding for the study, in cooperation with CS, RL, MO and FB. Recruitment of participants, data gathering and data analyses are coordinated by CSJ, under supervision of all other authors. CSJ wrote the manuscript, in cooperation with the other authors. All authors have read and approved the final manuscript.

## Authors’ information

Caroline Jonkman, MSc. is a child psychologist and PhD student at the department of Child and Adolescent Psychiatry at the AMC-Academic Medical Center (University of Amsterdam, the Netherlands).

Prof. Dr Carlo Schuengel is a professor at VU University and EMGO Institute for Health and Care Research and head of the department of Clinical Child and Family Studies and Special Education (Amsterdam, the Netherlands).

Dr Robert Lindeboom is a clinical epidemiologist at the department of Clinical Epidemiology and Biostatistics at the AMC-Academic Medical Center (University of Amsterdam, the Netherlands).

Dr Mirjam Oosterman is an assistant professor at VU University, department of Clinical Child and Family Studies and Special Education and EMGO Institute for Health and Care Research (Amsterdam, the Netherlands).

Prof. Dr Frits Boer is emeritus professor of the department of Child and Adolescent Psychiatry at the AMC-Academic Medical Center (University of Amsterdam, the Netherlands).

Dr Ramón JL Lindauer is a child and adolescent psychiatrist and family therapist, and head of the department of Child and Adolescent Psychiatry at the AMC-Academic Medical Center (University of Amsterdam, the Netherlands).
